# Real-Life Study of Patient Preference for Dupilumab or Revision Surgery for Recurrent Chronic Rhinosinusitis with Nasal Polyps

**DOI:** 10.3390/jpm14040338

**Published:** 2024-03-23

**Authors:** Katharina Gangl, David Tianxiang Liu, Tina Bartosik, Nicholas James Campion, Erich Vyskocil, Christian Albert Mueller, Birgit Knerer, Julia Eckl-Dorna, Sven Schneider

**Affiliations:** Department of Otorhinolaryngology, Head and Neck Surgery, Medical University of Vienna, Währinger Gürtel 18-20, A-1090 Vienna, Austria; katharina.gangl@meduniwien.ac.at (K.G.); david.liu@meduniwien.ac.at (D.T.L.); nicholas.campion@meduniwien.ac.at (N.J.C.); erich.vyskocil@meduniwien.ac.at (E.V.); christian.a.mueller@meduniwien.ac.at (C.A.M.); birgit.knerer@meduniwien.ac.at (B.K.); julia.eckl-dorna@meduniwien.ac.at (J.E.-D.)

**Keywords:** chronic rhinosinusitis, nasal polyps, endoscopic sinus surgery, Dupilumab, biologicals, quality of life

## Abstract

(1) Background: Chronic rhinosinusitis with nasal polyps (CRSwNP) has a high rate of recurrence in patients, despite therapy with local corticosteroids and functional endoscopic sinus surgery. Dupilumab, a recombinant monoclonal human IgG4 antibody directed against the IL-4 receptor α that inhibits both IL-4 and IL-13 signal transduction, is available for symptomatic therapy. Patient preference between repeated surgery and injection therapy with Dupilumab is not known. (2) Methods: Patients who had experienced at least one surgical intervention for nasal polyps and were treated with Dupilumab for at least 3 months completed a retrospective patient questionnaire. (3) Results: In a cohort of 75 previously operated CRSwNP patients, 91.5% preferred therapy with Dupilumab to repeated surgery for nasal polyps. Preference for Dupilumab in the subgroups of patients with concomitant Non-steroidal Anti-inflammatory Drugs Exacerbated Respiratory Disease (N-ERD) (*n* = 32), patients with concomitant asthma (*n* = 25), and patients without concomitant disease (*n* = 18) was 100%, 96%, and 72%, respectively. (4) Conclusions: Patient preference for Dupilumab over repeat surgery is strongest in previously operated CRSwNP patients with concomitant asthma or N-ERD, but remains very high in patients without concomitant disease.

## 1. Introduction

Chronic rhinosinusitis with nasal polyps (CRSwNP) is a common disease, affecting 1.9–4% of the European population [1,2,3,4,5]. Cases of CRSwNP in Europe are mostly dominated by type 2 inflammatory patterns characterized by eosinophil recruitment and activation, T-helper (Th2) type cytokine production, and IgE production [4]. In a subgroup of patients, CRSwNP is associated with asthma and Non-Steroidal Anti-inflammatory Drug (NSAID)-Exacerbated Respiratory Disease (N-ERD) [5,6,7].

Most frequently, CRSwNP patients suffer from nasal obstruction, loss of sense of smell (anosmia), runny nose, facial pain, and headaches. These symptoms can be assessed objectively by clinical and radiological examination [5]. As a consequence of these symptoms, the impact on the quality of life (QoL) of CRSwNP can be severe and has been compared to conditions such as chronic heart failure or asthma [8,9]. CRSwNP affects QoL on many levels and can also cause sleep, emotional, and mood disorders [10,11]. Assessment of patient symptoms via a questionnaire is increasingly used in routine practice to ascertain patient experience of symptom severity as a supplement to clinical and radiological examination [5]. The most common patient questionnaire used for CRS patients is the SNOT-22 (sino-nasal outcome test 22), which groups patient symptoms in four distinctive clusters regarding nasal symptoms, otologic/facial pain, emotional and sleep-related symptoms, but other questionnaires asking subjective and objective questions, such as the EQ-5D, are also in use for CRS patients [5,8,9,11]. Alarmingly, up to 41% of CRS patients recently reported uncontrolled symptoms in QoL assessments [12].

According to the latest guidelines, typical treatment modalities include intranasal corticosteroid application and saline irrigation, with additional short-term systemic corticosteroid bursts if symptoms are not sufficiently controlled. In cases with persistent symptoms, surgical treatment in the form of endoscopic sinus surgery (ESS) is performed.

Surgery has been well established in CRSwNP treatment and significant improvements in QoL and disease burden can be achieved rapidly. Many patients do well after surgery and experience substantial symptom relief [13]. However, a significant medical workload is associated with the surgical removal of polyps. Frequently performed under general anaesthesia, in-hospital stays are required and recuperation follows the procedure. Moreover, recurrences are seen at rates that increase with comorbidities such as asthma and N-ERD as risk factors [14,15,16,17,18]. In CRSwNP patients with type 2 inflammatory patterns suffering from additional asthma or N-ERD, recurrence rates can be as high as 60–90%, so many of these patients experience only short intervals of symptom relief [13,15,16,18].

Recently, several monoclonal antibodies have also become available for the treatment of CRSwNP as alternatives to surgery [2,5]. Dupilumab was approved by the European Medicines Agency and the U.S. Food and Drug Administration in 2019 for the treatment of CRSwNP, while already in use for other type 2 inflammatory diseases such as asthma and atopic dermatitis [19,20]. Dupilumab is a recombinant, human monoclonal antibody that binds to the IL-4α receptor and blocks both IL-4 and IL-13 signalling [2,5]. Efficacy in patients suffering from CRSwNP was demonstrated in Liberty Sinus nasal polyp studies that showed a significant and clinically relevant reduction of polyp size and improvements in disease-specific QoL, sense of smell, and comorbid asthma. Possible associated side effects reported were seldom and mild in nature and included fever, conjunctivitis, headaches, itching, and arthralgia [21].

In a recent study, Dupilumab improved CRSwNP in patients who had had previous surgery, with greater improvements in endoscopic outcomes in patients with shorter duration since the last surgery [22]. While the clinical benefit from both surgical and conservative treatment modalities is strong, patient preference is not yet known. In this study, we therefore evaluated patient responses in a cohort of recurrent CRSwNP who had experienced both, surgery and conservative treatment with Dupilumab.

## 2. Materials and Methods

### 2.1. Study Population

Patients aged over 18 years routinely treated for nasal polyps at the Department of Otorhinolaryngology of the Medical University of Vienna between 2019 and 2021 were included. Inclusion criteria were treatment with Dupilumab for at least 3 months and at least one ESS at the study centre or another hospital both inside and outside of Austria for nasal polyps. Mere polypectomies without opening of patient sinuses were excluded to the best of our knowledge by medical history, patient questioning, and admitting only procedures done under general anaesthesia, but the extent of previous ESS was not further assessed. Patients completed a retrospective patient questionnaire in German.

### 2.2. Ethics Approval and Consent to Participate

The ethical committee of the Medical University of Vienna approved the study (EK No 2452/2020), which was conducted according to the guidelines of the Declaration of Helsinki on Biomedical Research Involving Human Subjects. All patients provided written informed consent.

### 2.3. Questionnaire

The authors designed a questionnaire for use in this study. Apart from collecting general demographic information as well as medical history and treatment information, the questionnaire contained the following eight questions (Q):

(Q1) Post-operative pain: Patients were asked to record subjective, recalled post-operative pain on a visual analogue scale (VAS) of 100 mm. The result was then measured and recorded as a percentage.

(Q2) Duration of need for painkillers? (Q3) Duration of leave of absence from work necessitated by surgery? Q2 and Q3 had a distinct choice of four answers increasing in duration: One week, two weeks, up to a month, or longer.

(Q4) Polyp-free period after surgery? (Q5) Symptom-free period after surgery? Q4 and Q5 had a distinct choice of four answers increasing in duration: One month, several months, up to a year, or longer.

(Q6) Self-reported side effects of Dupilumab most bothersome to patients? Q6 had six possible answers: Fever, headache, eye inflammation, rash and itching, none, and other.

(Q7) Perceptions about the repeat injection of Dupilumab? Q7 had five possible answers: No problem, minimal problem, small problem, big problem, and worst possible problem.

(Q8) Preference between repeat operations and Dupilumab? Here, the choice of three answers was injection, operation, and indifferent.

### 2.4. Statistical Analysis

Excel (Microsoft for Mac, Version 16.59, Redmond, WA, USA) was used to record and administrate the patient data. GraphPad software (Version 6.0, San Diego, CA, USA) was used to calculate descriptive statistics (mean, median, range, percentage) and to create figures.

## 3. Results

### 3.1. Demographic Data

Patient characteristics are recorded in Table 1. Between April and September 2021, 100 patients receiving Dupilumab were approached to participate in the study and 88 patients returned a completed questionnaire. A cohort of 75 patients had experienced at least one verified endoscopic sinus surgery. This cohort of 75 patients had the following characteristics (Table 1): Median age was 52 (range 22–72). Gender distribution was 64% (*n* = 48) male and 36% (*n* = 27) female. The median length of therapy with Dupilumab at the time of the questionnaire was 7 months (range 3–15). Here, 25 patients (33.3%) were suffering from concomitant asthma, 32 patients (42.7%) were suffering from N-ERD, and 24% (*n* = 18) of the study group were without concomitant disease.

### 3.2. Number of Previous Operations

**Figure 1:**
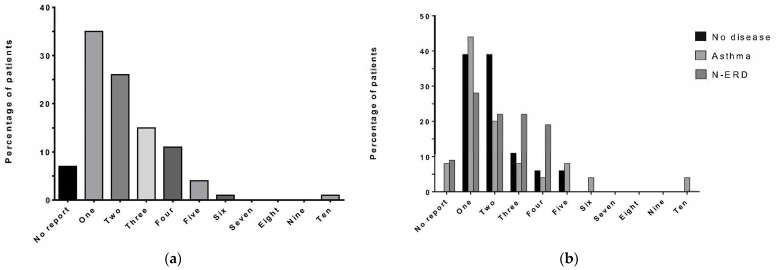
Number of previous operations. The number of previous operations experienced by study patients is displayed on the *x*-axis. The percentage of patients (**a**) in the entire cohort; (**b**) in different groups of no concomitant disease (*n* = 18, black), asthma (*n* = 25, light grey), and N-ERD (*n* = 32, dark grey) is displayed on the *y*-axis.

The median number of operations undergone by patients in the cohort prior to Dupilumab therapy was two (range 1–10). The majority of patients (*n* = 57, 76%) had between one and three previous surgeries, whereas 16% (*n* = 12) had 4–6 operations, and one patient had 10 previous surgeries (Figure 1a). The median number of operations for the three subgroups of patients without concomitant disease, with concomitant asthma, and with concomitant N-ERD was equally two (no concomitant disease, range 1–5 operations; asthma, range 1–10 operations; N-ERD, range 1–4 operations), and more than half of the patients had undergone 1–3 operations in each subgroup, Figure 1b.

### 3.3. Self-Reported Post-Operative Pain

On a VAS pain scale of 0% (no pain) to 100% (worst pain), the median of recalled post-operative pain was 44.7% (range 0–100) among the entire patient cohort. Median pain was 30.7% (0–100) in the subgroup with no concomitant disease, 46.8% (range 0–92.7) for the asthma subgroup, and 34.4% (range 0–95.9) for the N-ERD sub-group.

### 3.4. Self-Reported Side Effects and Duration of Success of Previous Operation

Approximately half of the patients in the entire cohort recalled a duration of pain and painkiller usage for a week, and only one patient reported the duration to be longer than a month. The results in the above-mentioned subgroups of patients without comorbidity, asthma, or N-ERD showed no striking deviation from this. The leave of absence from work due to surgery was below 2 weeks for approximately two-thirds of patients in the entire cohort. In the group of patients with no concomitant disease, the proportion of patients who stayed at home for up to two weeks was smaller, but more patients needed to stay at home for up to a month.

After operative intervention, a little less than half of the patients were free from symptoms for several months and did not suffer from polyps for several months. Only 13.3% (*n* = 57) of the entire study cohort were complaint-free for longer than a year, and only about a fifth of the entire cohort (*n* = 16; 21.3%) was polyp-free for longer than a year. The highest proportion of complaint-free patients for longer than a year (*n* = 6, 18.6%) was found in the N-ERD subgroup, while polyp-free patients for longer than a year were about a fifth of the sub-group in all subgroups. The results are summarized in Table 2.

### 3.5. Self-Reported Side Effects and Feelings towards Regular Injection of Dupilumab

**Figure 2:**
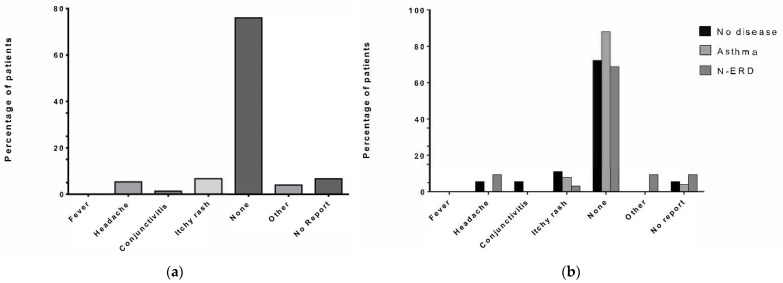
Number of previous operations. The number of previous operations experienced by study patients is displayed on the *x*-axis. (**a**) The percentage of patients in the entire cohort; (**b**) and of the different groups of no concomitant disease (*n* = 18, black), asthma (*n* = 25, light grey), and N-ERD (*n* = 32, dark grey) is displayed on the *y*-axis.

More than two-thirds of patients in the entire cohort (*n* = 57, 76%) did not report any side effects from Dupilumab injections. The most commonly reported side effect was an itchy rash in 7% of patients, followed by headaches in 4% of patients (Figure 2a). There were no obvious deviations from this distribution of side-effects in the abovementioned subgroups (Figure 2b).

In total, 56% of the entire patient cohort felt that regular injections were not seen as problematic, with an additional quarter of patients feeling the injections to be only a minimal problem (minimal problem 26%, minor problem 10%, major problem 1%, worst possible problem 0%, no report 7%). No obvious differences from that distribution were seen in the above-mentioned subgroups.

### 3.6. Patient Preference between Repeat Operations and Dupilumab Therapy

**Figure 3:**
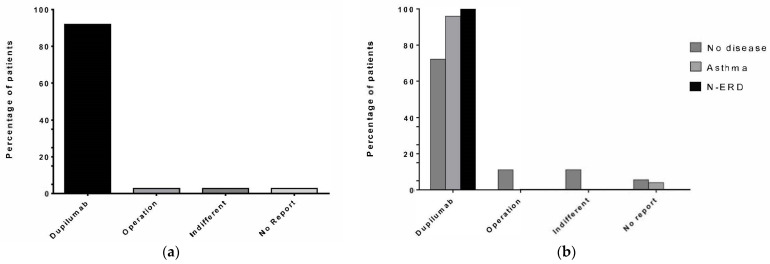
Number of previous operations. The number of previous operations experienced by study patients is displayed on the *x*-axis. (**a**) The percentage of patients in the entire cohort; (**b**) and of the different groups of no concomitant disease (*n* = 18, dark grey), asthma (*n* = 25, light grey), and N-ERD (*n* = 32, black) is displayed on the *y*-axis.

In the entire patient cohort, 91.5% (*n* = 69) of patients preferred Dupilumab to repeated surgery, whereas only 2.8% (*n* = 2) preferred surgery or stated no preference, respectively. All patients who preferred surgery or stated no preference had experienced one operation each. Results are shown in Figure 3a.

Patients with concomitant asthma (*n* = 25, 33.3% of the entire cohort) showed a 98% preference for Dupilumab, and only one patient who made no report (*n* = 1, 4%) was found in that group. Patients with concomitant N-ERD reported a 100% preference for Dupilumab. The smallest subgroup (*n* = 18, 24% of the entire cohort) were patients without concomitant disease, and the preference for Dupilumab in this subgroup was 72.2%. All patients who preferred the operation (*n* = 2, 11.1%) and all patients who had no preference (*n* = 2, 11.1%), as well as one patient who made no report (*n* = 1, 5.6%), were found in that group. Results for the subgroups are shown in Figure 3b.

## 4. Discussion

Surgery is an established mainstay in the treatment of CRSwNP. However, biologicals offer great benefits and have revolutionized therapeutic options. This is the first real-life study that aimed to evaluate patient preference in a cohort who experienced sinus surgery and treatment with a biological treatment, and we found that an overwhelming proportion of our cohort preferred Dupilumab treatment. Patient preference was stronger in patients suffering from concomitant asthma and strongest in patients suffering from N-ERD.

This study was designed as a retrospective questionnaire study in a previously operated patient cohort treated with Dupilumab at a tertiary centre and cannot be interpreted as a head-to-head comparison between ESS and Dupilumab. The aim of the study was to try and elucidate whether there was a patient preference in this patient cohort and—if possible—to find differences according to patient characteristics such as comorbidities in order to facilitate a more personalized approach to clinical medical decisions. There is no prejudice to surgery, which is a well-established therapy option in certain CRSwNP patients. Likewise, the aim of the study was not to demonstrate the efficiency of a proven therapy such as Dupilumab for CRSwNP but rather to record patient preference in our study cohort.

A certain bias is inherent to the design because patients having already failed surgery currently improving with Dupilumab might be more likely to answer the questionnaire in this specific study setting. However, the lived experience of recurrence is common in CRSwNP and this study displays a representative patient cohort seen in tertiary health care centres [23,24,25]. 100 patients received the questionnaire and the return rate of completed questionnaires was 88%, indicating a fairly comprehensive sample among all the patients that were approached.

It could be argued that some patients in our cohort experienced a recurrence of CRSwNP because of the insufficient extent of previous ESS and therefore indicated a preference for Dupilumab. Patients had been referred to the study centre for Dupilumab treatment by individual doctors in Eastern Austria and other Austrian hospitals and had been previously operated elsewhere. CT scans were not routinely performed prior to starting Dupilumab, and therefore we were unable to assess the full extent of previous ESS in the study cohort. Medical history of patients was taken, patients were questioned, and only procedures performed under full anaesthesia were included, so mere polypectomies without opening of the sinuses were excluded. However, referral to a tertiary centre indicates a population suffering from severe disease, which is underscored by the fact that many patients in the cohort were also suffering from asthma and/or NERD. Moreover, the median number of operations experienced by the entire study cohort was two, with some patients having undergone up to ten ESS, indicating severe and recurrent disease.

Even at a time when biologicals such as Dupilumab were not available, a substantial subset of CRSWNP patients preferred continued non-surgical management over ESS [26]. Worse levels in SNOT-22 scores were found to be the only predictor of the patient choice of surgery [27]. In previous studies, surgery for CRSwNP was reported to improve QoL assessed with the SNOT-22 questionnaire by 13.8 points, showing clinically relevant improvement considering the minimal clinically important difference is 8.9 points [14,28]. In the Liberty NP Sinus-24 study, Dupilumab was shown to reduce SNOT-22 values by 21.12 points [21] and in another study, SNOT-22 values were found to correlate with Total Polyp Score in CRSwNP [29]. Real-life studies showed even larger decreases in SNOT-22 values after 12 months of treatment [30,31]. Our result of strong patient preference for Dupilumab corresponds well with the bigger reduction in SNOT-22 values found in those studies. In contrast, another study found only slight differences between treatment with Dupilumab or ESS in the SNOT-22 scores of CRSwNP patients [32]. More specifically, patients treated with Dupilumab reported improved olfaction and decreased coughing, postnasal, and thick nasal drainage, whereas patients treated with ESS had a greater reduction in polyp burden [32]. However, even if SNOT-22 values after Dupilumab treatment are not overly different from values after ESS, it may be that the greater improvement for specific and unpleasant symptoms during Dupilumab therapy is more important to patient benefit perception than the actual polyp score reduction, and this could explain why patients in this study preferred Dupilumab. Recently, it was shown that olfaction scores may be worse after ESS surgery, and this may bias patient preference toward Dupilumab [33]. Furthermore, patient preference might also be due to the early recurrence of symptoms after surgery in contrast to the steady improvement of symptoms with Dupilumab [16]. As only 16 (21.3%) patients in the entire cohort were polyp-free for more than a year after the operations, this may be the reason for the stated patient preference.

At the time of the questionnaire, 13 patients (17.3%) in the entire study cohort reported experiencing side effects of Dupilumab, mostly rashes and itching, while the perceived recollected effects of previous surgery, mainly duration of post-operative pain, painkiller use, and median pain of 44.3% on a VAS of 100%, may have become ambiguous over time. The strong preference for Dupilumab is, however, still valid because it is the subjective memory of previous experiences that influences patient choice in real life.

Whereas therapy for asthma and atopic dermatitis with Dupilumab is well established [34,35], treatment of nasal polyps with Dupilumab was only approved in 2019. The median period of analysis in our study is 7 months, and it remains to be seen whether patient preference will evolve and change over time. However, acceptance of long-term therapies is common in patients receiving other biologicals for other indications. Long-term therapy for two years with Dupilumab has been demonstrated for CRSwNP [31] and even longer for other indications of Dupilumab [36]. The fact that 82% of patients in our study considered the injection no problem or a minimal problem further underlines this preference.

Longer treatment with Dupilumab will also increase treatment costs, and it has been argued that initial and repeat ESS are more cost-effective than Dupilumab treatment in an unselected patient cohort [37]. Appropriate patient selection, therefore, is crucial. More than two-thirds of the study patients were suffering from asthma or N-ERD, and in those patient groups, preference for Dupilumab was more pronounced than in patients without concomitant disease, even if the preference for Dupilumab remained remarkably high at 72% in the smaller cohort of patients without asthma or N-ERD. Possibly, patients with concomitant asthma or N-ERD experienced a greater improvement in general Qol, which may be seen as a confounding factor influencing patient preference for Dupilumab. On the other hand, and considering patient preference as demonstrated in this study, concomitant asthma or N-ERD may influence the decision whether to treat patients with CRSwNP recurrence with repeated ESS surgery or with Dupilumab, as only Dupilumab will have a favourable influence on these concomitant diseases.

Guidelines from the European Forum for Research and Education in Allergy and Airway Diseases and the European Position Paper on Rhinosinusitis and Nasal Polyps (EPOS) incorporate initial surgical therapy before the selection of biological treatment [2,5]. In EPOS guidelines, prior surgery is required before biological treatment should be considered, but neither set of guidelines excludes a second surgical intervention [5]. Our result showing a strong patient preference for subcutaneous administration of a biological in recurrent CRSwNP after surgery is within the scope of these guidelines.

Although patients in our cohort decided strongly for treatment with Dupilumab, especially patients experiencing a first recurrence of nasal polyps may benefit equally from a second surgical intervention. This is underpinned by the fact that the patients in the study cohort who expressed no preference or preferred surgery had only undergone one surgical intervention each. On the other hand, the stronger preference for Dupilumab in patients with concurrent asthma or N-ERD and the general improvement of QoL that can be inferred for that cohort suggest that recurrent CRSwNP patients suffering from concurrent N-ERD or asthma will benefit most from treatment with a biological like Dupilumab.

In addition, cases that would benefit most from primary antibody therapy need to be defined. Especially N-ERD patients, who have a very high risk of recurrence, and patients with a high risk for surgery may benefit from primary therapy with a biological. In this context, as well, it is not surprising that the N-ERD subgroup expressed 100% preference for Dupilumab in our study.

The clinical implication that follows from our study is a stratification for clinical decision making taking into account patient preference. If a previously operated patient presents again with a recurrence of CRSwNP, note should be taken of patient comorbidities. Patients without comorbidities may benefit from another round of surgery, as they do not unequivocally prefer biologics therapy. Biologics therapy may be considered more favourably for patients suffering from asthma, and most favourably for patients suffering from N-ERD.

## 5. Conclusions

In conclusion, as a result of the first real-life questionnaire study, previously operated CRSwNP patients overwhelmingly preferred therapy with Dupilumab to repeat surgical intervention. The preference was stronger in patients suffering from concurrent asthma and strongest in patients suffering from N-ERD, indicating that patients with these concomitant diseases benefit most.

## Figures and Tables

**Table 1:** Patient characteristics.

Patient Characteristic	Number (%)
Patients	75 (100)
Sex	
Male	48 (64)
Female	27 (36)
Age	
Median, years	52
Range, years	22–74
Mean treatment duration	
Median, months	7
Range, months	3–15
Comorbidities	
None	18 (24.0)
Asthma	25 (33.3)
N-ERD	32 (42.7)

**Table 2:** Side Effects of Operations.

	Number (%)			
	Entire Cohort	No Comorbidity	Asthma	N-ERD
Postoperative Duration of pain				
One week	35 (46.7)	9 (50.0)	11 (44.0)	15 (46.9)
Two weeks	21 (28.0)	3 (16. 7)	7 (28.0)	11 (34.4)
Up to a month	10 (13.3)	4 (22.2)	4 (16.0)	2 (6.25)
Longer	1 (1.3)	1 (5.6)	0	0
No report	8 (10.7)	1 (5.6)	3 (12.0)	4 (12.5)
Postoperative need for painkillers				
One week	47 (62.7)	8 (55.6)	20 (80.0)	17 (53.1)
Two weeks	12 (16)	5 (27.8)	2 (8.0)	5 (15.6)
Up to a month	3 (4)	2 (11.1)	0	1 (3.1)
Longer	1(1.3)	0	0	1 (3.1)
No report	12 (16)	1 (5.6)	3 (12.0)	8 (25.0)
Absence from work after operation				
One week	31 (41.3)	6 (33.3)	12 (48.0)	13 (40.6)
Two weeks	22 (29.3)	2 (11.1)	8 (32.0)	12 (37.5)
Up to a month	13 (17.3)	5 (27.8)	2 (8.0)	6 (18.75)
Longer	0 (0)	0	0	0
No report	9 (12)	5 (27.8)	3 (12.0)	1 (3.1)
Complaint-free period after operation				
One month	19 (25.3)	4 (22.2)	7 (28.0)	8 (25.0)
Two months	31 (41.3)	10 (55.6)	10 (40.0)	11 (34.4)
Up to a year	10 (13.3)	1 (5.6)	4 (16.0)	5 (15.6)
Longer	10 (13.3)	2 (11.1)	2 (8.0)	6 (18.75)
No report	5 (6.7)	1 (5.6)	2 (8.0)	2 (6.25)
Polyp-free period after operation				
One month	15 (20)	4 (22.2)	6 (24.0)	5 (15.6)
Two months	32 (42.7)	9 (50.0)	8 (32.0)	15 (46.9)
Up to a year	7 (9.3)	0	3 (12.0)	4 (12.5)
Longer	16 (21.3)	4 (22.2)	5 (20.0)	7 (21.9)
No report	5 (6.7)	1 (5.6)	3 (12.0)	1 (3.13)

## Data Availability

The data analysed in the study are available upon request to the corresponding author.
